# Supplementary material for: Pediatric Infections by Human mastadenovirus C Types 2, 89, and a Recombinant Type Detected in Japan between 2011 and 2018
Source: Viruses. 2019 Dec 6;11(12):1131. doi: 10.3390/v11121131 (PMC6950085; doi:10.3390/v11121131)
Supplement: Supplementary file 1 [file viruses-11-01131-s001.pdf]

Supplementary Materials

Table S1. Partial sequences of penton base- and hexon-coding regions from 100 Ad positive samples.

		Accession Numbers			Clustering Type			
**Strain**	**Year**	**Penton Base**	**Hexon**	**Complete Genome**	**Penton Base**	**Similarity (%)**	**Hexon**	**Similarity (%)**
**K10-037-2012**	2012	LC498892	LC499891	-	Ad1 (AC_000017)	99.8	Ad2 (AC_000007)	98.3
**K11-042-2012**	2012	LC498893	LC499892	-	Ad1 (AC_000017)	99.8	Ad2 (AC_000007)	99.9
**K13-060-2012**	2012	LC498895	LC499894	-	Ad1 (AC_000017)	99.8	Ad2 (AC_000007)	99.9
**K16-074-2012**	2012	LC498898	LC499897	-	Ad1 (AC_000017)	99.7	Ad2 (AC_000007)	99.7
**K17-075-2012**	2012	LC498899	LC499898	-	Ad1 (AC_000017)	99.8	Ad2 (AC_000007)	99.5
**K18-083-2012**	2012	LC498900	LC499899	-	Ad1 (AC_000017)	99.7	Ad2 (AC_000007)	100.0
**K3-022-2012**	2012	LC498885	LC499884	-	Ad1 (AC_000017)	99.8	Ad2 (AC_000007)	99.0
**K4-026-2012**	2012	LC498886	LC499885	-	Ad1 (AC_000017)	99.8	Ad2 (AC_000007)	99.9
**K5-029-2012**	2012	LC498887	LC499886	-	Ad1 (AC_000017)	99.8	Ad2 (AC_000007)	99.5
**K6-030-2012**	2012	LC498888	LC499887	-	Ad1 (AC_000017)	99.8	Ad2 (AC_000007)	99.9
**K8-032-2012**	2012	LC498890	LC499889	-	Ad1 (AC_000017)	99.8	Ad2 (AC_000007)	99.9
**K21-088-2013**	2013	LC498903	LC499902	-	Ad1 (AC_000017)	99.8	Ad2 (AC_000007)	99.9
**K22-098-2013**	2013	LC498904	LC499903	-	Ad1 (AC_000017)	99.8	Ad2 (AC_000007)	99.9
**K23-099-2013**	2013	LC498905	LC499904	-	Ad1 (AC_000017)	99.8	Ad2 (AC_000007)	99.6
**K24-100-2013**	2013	LC498906	LC499905	-	Ad1 (AC_000017)	99.8	Ad2 (AC_000007)	99.4
**K25-103-2013**	2013	LC498907	LC499906	-	Ad1 (AC_000017)	99.8	Ad2 (AC_000007)	99.9
**K26-105-2013**	2013	LC498908	LC499907	-	Ad1 (AC_000017)	99.8	Ad2 (AC_000007)	99.5
**K28-110-2013**	2013	LC498910	LC499909	-	Ad1 (AC_000017)	99.8	Ad2 (AC_000007)	99.0
**K29-111-2013**	2013	LC498911	LC499910	-	Ad1 (AC_000017)	99.8	Ad2 (AC_000007)	99.1
**K31-117-2013**	2013	LC498913	LC499912	-	Ad1 (AC_000017)	99.8	Ad2 (AC_000007)	99.6
**K32-119-2013**	2013	LC498914	LC499913	-	Ad1 (AC_000017)	99.8	Ad2 (AC_000007)	99.6
**K33-124-2013**	2013	LC498915	LC499914	-	Ad1 (AC_000017)	99.8	Ad2 (AC_000007)	99.1
**K34-131-2013**	2013	LC498916	LC499915	-	Ad1 (AC_000017)	99.8	Ad2 (AC_000007)	100.0
**K36-166-2013**	2013	LC498918	LC499917	-	Ad1 (AC_000017)	99.8	Ad2 (AC_000007)	99.9
**K37-170-2013**	2013	LC498919	LC499918	-	Ad1 (AC_000017)	99.9	Ad2 (AC_000007)	98.8
**K38-178-2014**	2014	LC498920	LC499919	-	Ad1 (AC_000017)	99.8	Ad2 (AC_000007)	99.2
**K39-190-2014**	2014	LC498921	LC499920	-	Ad1 (AC_000017)	99.7	Ad2 (AC_000007)	99.0
**K40-191-2014**	2014	LC498922	LC499921	-	Ad1 (AC_000017)	99.7	Ad2 (AC_000007)	99.7
**K41-193-2014**	2014	LC498923	LC499922	-	Ad1 (AC_000017)	99.7	Ad2 (AC_000007)	99.0
**K42-194-2014**	2014	LC498924	LC499923	-	Ad1 (AC_000017)	99.7	Ad2 (AC_000007)	99.7
**K43-195-2014**	2014	LC498925	LC499924	-	Ad1 (AC_000017)	99.8	Ad2 (AC_000007)	99.2
**K44-197-2014**	2014	LC498926	LC499925	-	Ad1 (AC_000017)	99.9	Ad2 (AC_000007)	99.2
**K45-211-2014**	2014	LC498927	LC499926	-	Ad1 (AC_000017)	99.8	Ad2 (AC_000007)	99.9
**K46-213-2014**	2014	LC498928	LC499927	-	Ad1 (AC_000017)	99.8	Ad2 (AC_000007)	99.2
**K47-215-2014**	2014	LC498929	LC499928	-	Ad1 (AC_000017)	99.7	Ad2 (AC_000007)	99.9
**K48-216-2014**	2014	LC498930	LC499929	-	Ad1 (AC_000017)	99.8	Ad2 (AC_000007)	99.9
**K49-218-2014**	2014	LC498931	LC499930	-	Ad1 (AC_000017)	99.8	Ad2 (AC_000007)	99.9
**K50-219-2014**	2014	LC498932	LC499931	-	Ad1 (AC_000017)	99.8	Ad2 (AC_000007)	99.0
**K51-227-2014**	2014	LC498933	LC499932	-	Ad1 (AC_000017)	99.8	Ad2 (AC_000007)	99.9
**K52-234-2014**	2014	LC498934	LC499933	-	Ad1 (AC_000017)	99.8	Ad2 (AC_000007)	99.1
**K53-243-2014**	2014	LC498935	LC499934	-	Ad1 (AC_000017)	99.7	Ad2 (AC_000007)	98.6
**K54-249-2014**	2014	LC498936	LC499935	-	Ad1 (AC_000017)	99.7	Ad2 (AC_000007)	99.9
**K56-262-2015**	2015	LC498938	LC499937	-	Ad1 (AC_000017)	99.7	Ad2 (AC_000007)	99.9
**K58-274-2015**	2015	LC498940	LC499939	-	Ad1 (AC_000017)	99.7	Ad2 (AC_000007)	99.5
**K59-280-2015**	2015	LC498941	LC499940	-	Ad1 (AC_000017)	99.8	Ad2 (AC_000007)	99.0
**K60-283-2015**	2015	LC498942	LC499941	-	Ad1 (AC_000017)	99.8	Ad2 (AC_000007)	99.9
**K61-286-2015**	2015	LC498943	LC499942	-	Ad1 (AC_000017)	99.9	Ad2 (AC_000007)	98.9
**K63-293-2015**	2015	LC498945	LC499944	-	Ad1 (AC_000017)	99.8	Ad2 (AC_000007)	99.6
**K64-294-2015**	2015	LC498946	LC499945	-	Ad1 (AC_000017)	99.8	Ad2 (AC_000007)	100.0
**K65-300-2015**	2015	LC498947	LC499946	-	Ad1 (AC_000017)	99.8	Ad2 (AC_000007)	99.9
**K66-324-2015**	2015	LC498948	LC499947	-	Ad1 (AC_000017)	99.8	Ad2 (AC_000007)	99.4
**K100-390-2016**	2016	LC498982	LC499981	-	Ad1 (AC_000017)	99.9	Ad2 (AC_000007)	99.0
**K69-345-2016**	2016	LC498951	LC499950	-	Ad1 (AC_000017)	99.9	Ad2 (AC_000007)	99.5
**K71-349-2016**	2016	LC498953	LC499952	-	Ad1 (AC_000017)	99.7	Ad2 (AC_000007)	99.9
**K72-353-2016**	2016	LC498954	LC499953	-	Ad1 (AC_000017)	99.7	Ad2 (AC_000007)	98.8
**K73-359-2016**	2016	LC498955	LC499954	-	Ad1 (AC_000017)	99.8	Ad2 (AC_000007)	100.0
**K74-364-2016**	2016	LC498956	LC499955	-	Ad1 (AC_000017)	99.8	Ad2 (AC_000007)	99.9
**K75-368-2016**	2016	LC498957	LC499956	-	Ad1 (AC_000017)	99.7	Ad2 (AC_000007)	99.9
**K76-382-2016**	2016	LC498958	LC499957	-	Ad1 (AC_000017)	99.8	Ad2 (AC_000007)	98.9
**K81-400-2017**	2017	LC498963	LC499962	-	Ad1 (AC_000017)	99.8	Ad2 (AC_000007)	99.9

**K82-409-2017**	2017	LC498964	LC499963	-	Ad1 (AC_000017)	99.9	Ad2 (AC_000007)	99.0
**K83-411-2017**	2017	LC498965	LC499964	-	Ad1 (AC_000017)	99.9	Ad2 (AC_000007)	99.0
**K84-419-2017**	2017	LC498966	LC499965	-	Ad1 (AC_000017)	99.8	Ad2 (AC_000007)	99.9
**K85-422-2017**	2017	LC498967	LC499966	-	Ad1 (AC_000017)	99.8	Ad2 (AC_000007)	100.0
**K86-430-2017**	2017	LC498968	LC499967	-	Ad1 (AC_000017)	99.6	Ad2 (AC_000007)	99.7
**K87-452-2017**	2017	LC498969	LC499968	-	Ad1 (AC_000017)	99.8	Ad2 (AC_000007)	100.0
**K91-482-2017**	2017	LC498973	LC499972	-	Ad1 (AC_000017)	99.9	Ad2 (AC_000007)	99.9
**K93-490-2018**	2018	LC498975	LC499974	-	Ad1 (AC_000017)	99.7	Ad2 (AC_000007)	99.9
**K94-493-2018**	2018	LC498976	LC499975	-	Ad1 (AC_000017)	99.8	Ad2 (AC_000007)	99.9
**K96-509-2018**	2018	LC498978	LC499977	-	Ad1 (AC_000017)	99.5	Ad2 (AC_000007)	99.9
**K97-512-2018**	2018	LC498979	LC499978	-	Ad1 (AC_000017)	99.5	Ad2 (AC_000007)	99.9
**K98-526-2018**	2018	LC498980	LC499979	-	Ad1 (AC_000017)	99.8	Ad2 (AC_000007)	99.9
**K99-529-2018**	2018	LC498981	LC499980	-	Ad1 (AC_000017)	99.7	Ad2 (AC_000007)	100.0
**K1-006-2012**	2012	LC498883	LC499882	-	Ad6 (FJ349096)	98.6	Ad2 (AC_000007)	99.7
**K12-044-2012**	2012	LC498894	LC499893	-	Ad6 (FJ349096)	98.6	Ad2 (AC_000007)	99.7
**K14-065-2012**	2012	LC498896	LC499895	-	Ad6 (FJ349096)	98.6	Ad2 (AC_000007)	99.7
**K15-067-2012**	2012	LC498897	LC499896	-	Ad6 (FJ349096)	98.6	Ad2 (AC_000007)	99.1
**K19-085-2012**	2012	LC498901	LC499900	LC504572	Ad6 (FJ349096)	98.6	Ad2 (AC_000007)	99.7
**K20-087-2013**	2013	LC498902	LC499901	-	Ad6 (FJ349096)	98.6	Ad2 (AC_000007)	99.7
**K30-114-2013**	2013	LC498912	LC499911	-	Ad6 (FJ349096)	98.6	Ad2 (AC_000007)	99.7
**K55-255-2014**	2014	LC498937	LC499936	-	Ad6 (FJ349096)	98.6	Ad2 (AC_000007)	98.9
**K57-264-2015**	2015	LC498939	LC499938	-	Ad6 (FJ349096)	98.6	Ad2 (AC_000007)	99.7
**K68-341-2016**	2016	LC498950	LC499949	-	Ad6 (FJ349096)	98.6	Ad2 (AC_000007)	99.2
**K70-348-2016**	2016	LC498952	LC499951	-	Ad6 (FJ349096)	98.6	Ad2 (AC_000007)	99.1
**K79-393-2016**	2016	LC498961	LC499960	-	Ad6 (FJ349096)	98.6	Ad2 (AC_000007)	99.7
**K80-397-2017**	2017	LC498962	LC499961	-	Ad6 (FJ349096)	98.6	Ad2 (AC_000007)	99.7
**K88-458-2017**	2017	LC498970	LC499969	-	Ad89 (MH121097)	99.8	Ad89 (MH121097)	99.0
**K89-464-2017**	2017	LC498971	LC499970	-	Ad89 (MH121097)	99.8	Ad89 (MH121097)	99.9
**K2-008-2012**	2012	LC498884	LC499883	-	Ad89 (MH121114)	99.8	Ad89 (MH121097)	99.7
**K7-031-2012**	2012	LC498889	LC499888	-	Ad89 (MH121114)	99.8	Ad89 (MH121097)	99.2
**K9-033-2012**	2012	LC498891	LC499890	-	Ad89 (MH121114)	99.5	Ad2 (AC_000007)	99.9
**K27-108-2013**	2013	LC498909	LC499908	-	Ad89 (MH121114)	99.8	Ad89 (MH121097)	99.9
**K35-151-2013**	2013	LC498917	LC499916	-	Ad89 (MH121114)	99.6	Ad2 (AC_000007)	99.9
**K62-291-2015**	2015	LC498944	LC499943	-	Ad89 (MH121114)	99.8	Ad89 (MH121097)	99.9
**K67-339-2016**	2016	LC498949	LC499948	LC504573	Ad89 (MH121114)	99.8	Ad89 (MH121097)	99.2
**K77-386-2016**	2016	LC498959	LC499958	-	Ad89 (MH121114)	99.9	Ad89 (MH121097)	99.9
**K78-387-2016**	2016	LC498960	LC499959	-	Ad89 (MH121114)	99.9	Ad89 (MH121097)	99.9
**K90-470-2017**	2017	LC498972	LC499971	-	Ad89 (MH121114)	99.6	Ad2 (AC_000007)	99.9
**K92-488-2018**	2018	LC498974	LC499973	-	Ad89 (MH121114)	99.8	Ad89 (MH121097)	99.7
**K95-504-2018**	2018	LC498977	LC499976	-	Ad89 (MH121114)	99.8	Ad89 (MH121097)	99.5

Table S2. Clinical Manifestations.

Clinical manifestations	Ad-2	Ad-89	Recombinant type	P-value*
**Total Cases**	71	13	13	
**Age (month)**	20 [17-31]	22 [16.5-34.5]	22 [14.5-43.5]	0.88
**Gender (male: female)**	41:30	7:6	5:8	0.49
**Maximum fever (°C)**	39.6 [39.3-40.0]	39.5 [39.3-40.0]	39.7 [39.0-40.1]	0.94
**Duration of fever (days)**	3 [3-4]	4 [3-5]	3 [2.5-4]	0.27
**Ocular symptoms**	2	0	0	1.00
**White Blood Cells (WBC) ×1000+**	15.2 [12.6-19.0]	13.0 [9.7-18.2]	15.3 [12.4-16.7]	0.39
**C-reactive Protein (CRP)**	2.3 [1.5-3.4]	2.5 [1.0-3.7]	2.3 [1.3-3.6]	1.00
**Exudative tonsillitis**	31	5	8	0.44
**Lower respiratory tract infection**	23	6	3	0.50

Continuous variables are indicated by the median [interquartile range]

* Fisher's exact test for dichotomous variables (ocular symptom, exudative tonsillitis and lower respiratory tract infection) and Kruskal-Wallis test for continuous variables (age, maximum fever, fever lasting days, WBC and CRP). P-value < 0.05 was considered as significant.

+ WBC are counted on the thousands
